# Robotic endoscope with double-balloon and double-bend tube for colonoscopy

**DOI:** 10.1038/s41598-023-37566-3

**Published:** 2023-06-28

**Authors:** Toshihiro Takamatsu, Yuto Endo, Ryodai Fukushima, Tatsuki Yasue, Kensuke Shinmura, Hiroaki Ikematsu, Hiroshi Takemura

**Affiliations:** 1 Exploratory Oncology Research and Clinical Trial Center, National Cancer Center, 6-5-1, Kashiwanoha, Kashiwa, Chiba, 277-8577 Japan; 2 Research Institute for Biomedical Sciences, Tokyo University of Science, Noda, Chiba, Japan; 3 Department of Mechanical Engineering, Tokyo University of Science, Noda, Chiba, Japan; 4 Department of Gastroenterology and Endoscopy, National Cancer Center Hospital East, Kashiwa, Chiba, Japan

**Keywords:** Colonoscopy, Biomedical engineering, Mechanical engineering

## Abstract

The insertion of conventional colonoscopes can sometimes cause patients to experience pain during the procedure owing to the stretching of the mesentery. In this study, a prototype of a robotic colonoscope with a double-balloon and double-bend tube based on the conventional double-balloon endoscope was developed to simplify insertion and prevent the overstretching of the colon. Both the outer and inner tubes were confirmed to be free from interference from wires and sheaths. Additionally, all functions such as tip bending, inflation and deflation of the balloons, and actuator-driven pulling and pushing of the inner tube were operated properly. During the insertion test, the device could be reached the cecum of a colon model in approximately 442 s when operated by a non-medical operator. In addition, the device did not overstretch the colon model, thereby suggesting that the insertion mechanism can follow the shape of the colon model. As a result, the developed mechanism has the potential to navigate through a highly-bent colon without overstretching.

Presently, colorectal cancer is the second leading cause of death worldwide^1^. Therefore, screening tests, such as a colonoscopy and fecal occult blood test (FOBT), are performed to reduce the mortality and incidence of colorectal cancer^2–5^. The patients who receive a positive result from the FOBT test eventually require a colonoscopy to confirm the presence of lesions. Although colonoscopy is the gold standard for detecting and diagnosing colorectal cancer, this procedure is considered to be burdensome to patients because of the pain they experience during the insertion of the colonoscope^6^. The pain is caused by the overstretching of the mesentery, and several factors such as gender, body mass index, and postoperative adhesions can determine the pain sensation of the patient^7–9^. Additionally, studies suggest that the insertion technique used by the endoscopist is particularly important^8,10,11^. Ideally, all endoscopists should be proficient in these insertion techniques; however, these techniques have a learning curve^12,13^. Therefore, a new colonoscope that is painless for the patient and easy to insert into the colon is required.

Insertion techniques that simplify colonoscopy have been investigated in previous studies. For example, a soft robotic structure, a structure with a propulsion mechanism at the tip, and an endoscope manipulated by a robot have been reported^14–16^. Moreover, advanced research by Era Endoscopy has produced the Endotics® System, which has been verified for use in clinical practice^17^. Although this system reduces the pain felt by the patient in comparison with conventional colonoscopes, the issue of high insertion time or incomplete colonoscopy persists.

Balloon endoscopes, which allow assisted insertion, have been used clinically^18,19^. A balloon endoscope is inserted by fixing the freely moving intestinal tract using an inflated balloon and operating the endoscope back and forth through an outer tube. This endoscope is often used for the diagnosis of small bowel diseases^20^, and its use in colonoscopy has been investigated^21^. One study reported that total colonoscopy was possible using a double-balloon endoscope (DBE) in patients who previously underwent incomplete colonoscopies^21^. This implies that the DBE holds the potential as a key technology for simplifying standard colonoscopy procedures. However, conventional DBEs face certain challenges stemming from the absence of a bend function at the tip of the outer tube and the lack of an electrical push/pull mechanism for the endoscope: (i) When the intestinal tract is highly bent, as shown in Fig. 1A, the force during insertion is transmitted only in the coaxial direction even if the tip of the colonoscope is curved in the direction of insertion. Therefore, the intestinal tract continues to be stretched owing to simple operations such as bending the tip and pushing or pulling the endoscope. (ii) Often two persons are required for performing the colonoscopy because operating the endoscope and outer tube is complicated.

**Figure 1:**
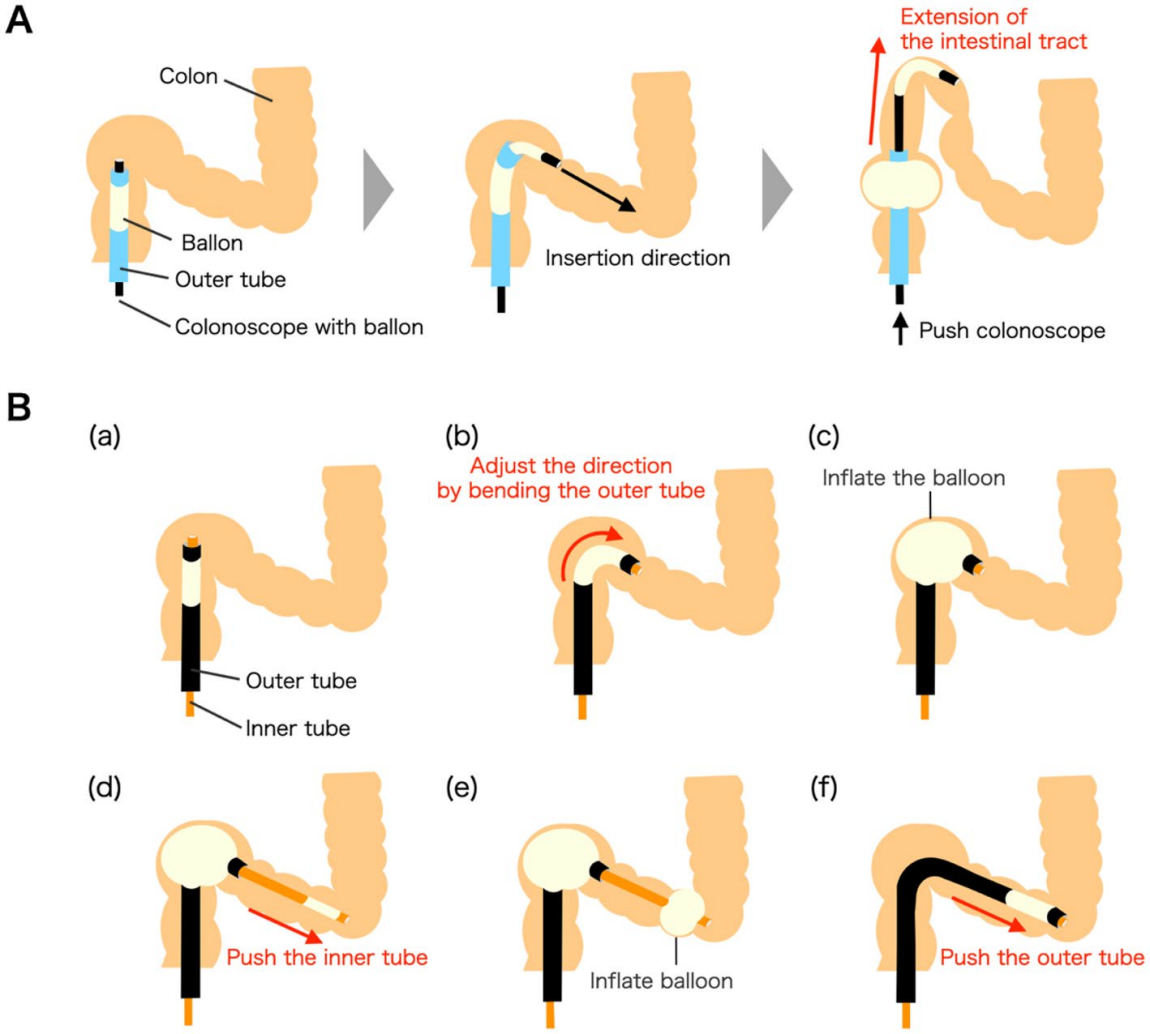
(**A**) Problems associated with conventional double-balloon endoscopes (**B**) Principle of device insertion based on our hypothesis. (**a**) The tip of the device reaches the bend in the colon. (**b**) The outer tube is bent to adjust the insertion direction. (**c**) The intestinal tract is fixed by the balloon attached to the outer tube. (**d**) The inner tube is pushed out. (**e**) The intestinal tract is fixed by the balloon attached to the inner tube. (**f**) The outer tube is pushed out and the inner tube is retracted.

To overcome the aforementioned problems, we propose a robotic endoscope based on the DBE. The outer and inner tubes of the proposed device are independently manipulated, and they can be operated electrically via a controller. More specifically, the bending of the outer and inner tubes, pulling and pushing of the inner tube, and inflation and deflation of each balloon can be operated using a controller. The mechanism of the proposed device is characterized by the tip of the inner tube being pushed out to the off-axis according to the direction of the tip of the outer tube. This insertion technique is expected to prevent the intestinal tract from being overstretched during the procedure. By pushing and pulling the outer tube with one hand and operating the controller using the other hand, a single individual can perform the colonoscopy. Therefore, the proposed endoscopic motion is expected to simplify insertion and furthermore automatic insertion during general endoscopic procedures such as screening and surveillance.

To the best of our knowledge, a device that with an electrically controllable double-balloon and double-bend tube has not been reported in the literature thus far, and the need for an easy insertion method for a highly bent colon persists. In this study, the robotic endoscope that can perform the aforementioned operations was developed and its successful insertion into a colon model was demonstrated.

## Result

### Verification of the manipulation of the device

**Figure 2:**
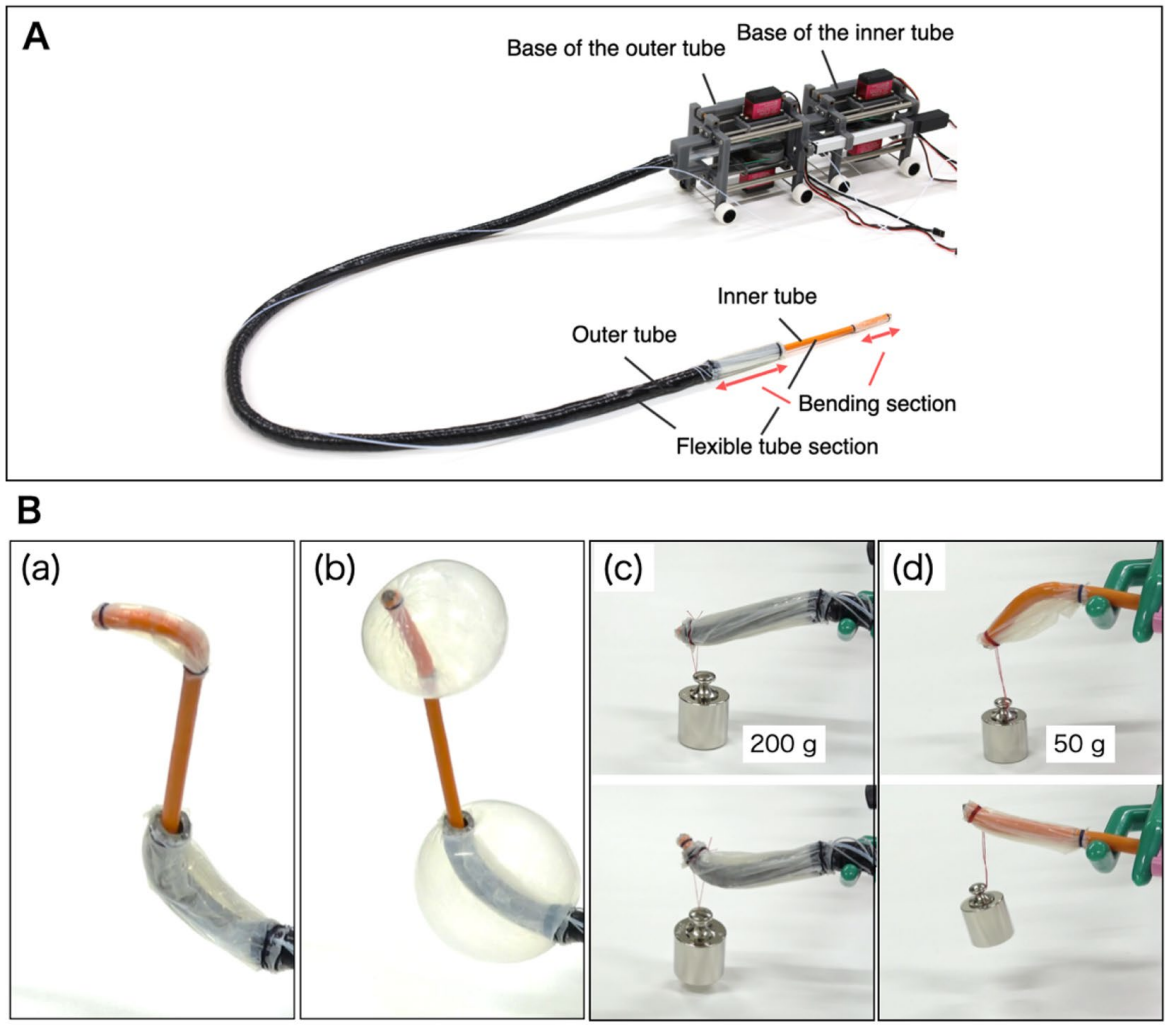
(**A**) Assembled device. (**B**) Motion and payload of the device. (**a**) S-shaped bending. (**b**) Double balloon inflation. (**c**) Payload of the outer tube. (**d**) Payload of the inner tube.

Figure 2A shows the developed device. The ability of the controller to control the bending of the outer and inner tubes, inflation and deflation of the balloons attached to the outer and inner tubes, and pushing and pulling of the inner tube was confirmed (see Supplementary Video S1 online). The length and diameter of the portion of the outer tube meant for insertion are approximately 114 cm and 17.0 mm, respectively. The maximum bending angles of the outer and inner tubes were approximately 90 and 180°, respectively. As shown in Fig. 2Ba, S-shaped bending that cannot be achieved by manipulating a conventional DBE, is achieved using the proposed device. As shown in Fig. 2Bb, the balloons inflate to a diameter of approximately 7 cm, which is sufficient for intestinal fixation. In addition, the tips of the outer and inner tubes are capable of lifting a maximum load of 200 and 50 g, respectively, as shown in Fig. 2Bc,d. The bending of the outer and inner tubes was constricted by wire traction when the servomotors remained fixed to the base and the flexible sections of the tube were highly bent. However, changing the position of the servomotors allows the bending sections to bend up, down, left, and right even when the flexible sections are highly bent, as shown in Fig. 4.

### Insertion test using a colon model

**Figure 3:**
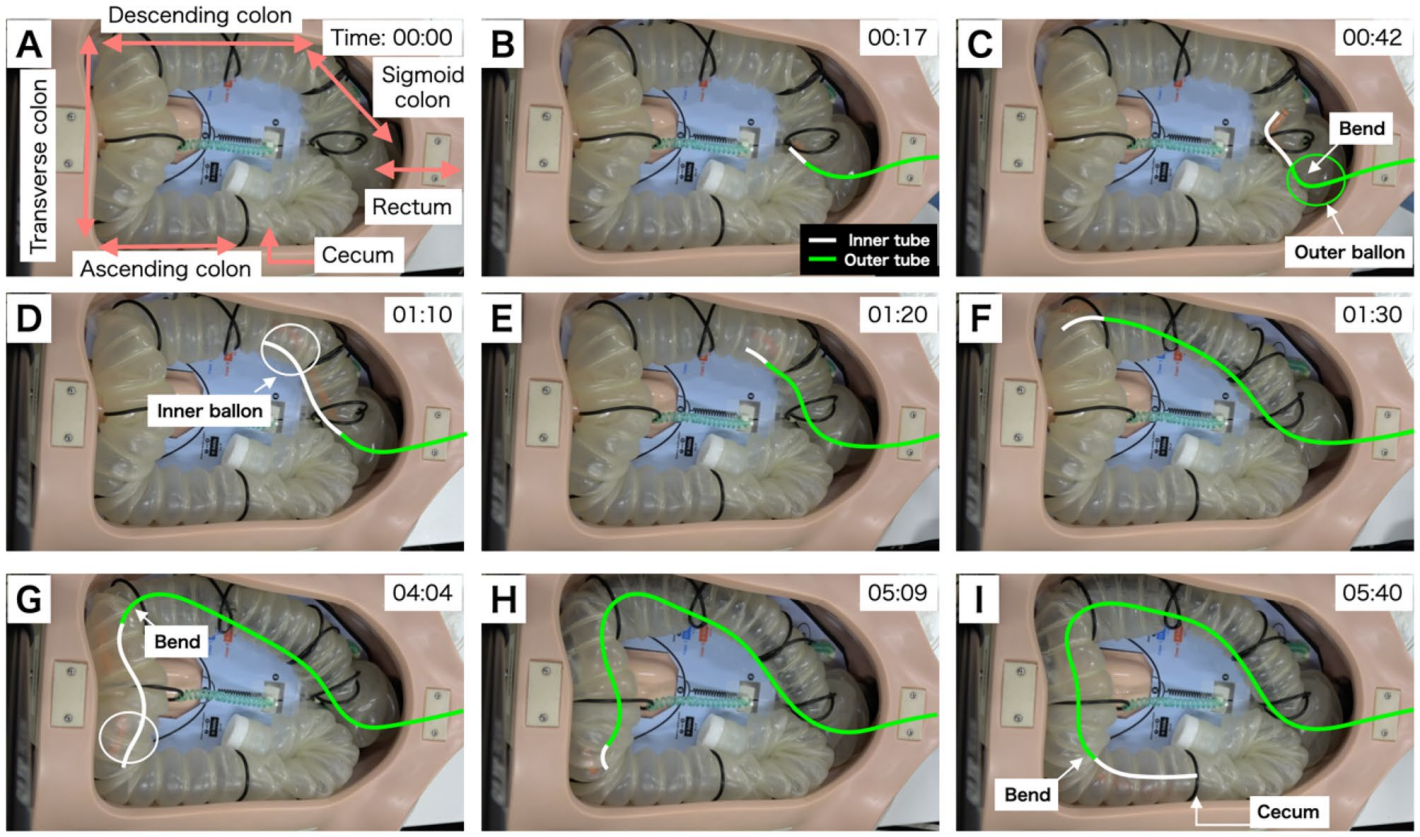
Insertion of the device into the colon model (Case 1); white line: inner tube, green line: outer tube, white circle: inflation of the balloon attached to the inner tube, green circle: inflation of the balloon attached to the outer tube. (**A**) The device tip is inserted into the anus. (**B**) The outer tube reaches the entrance of the sigmoid colon. (**C**) The inner tube is pushed out to pass through the sigmoid colon. (**D**) The balloon attached to the inner tube is inflated to fix the descending colon. (**E**) Initial state of the device in the descending colon. (**F**) Outer tube reaches the entrance of the transverse colon. (**G**) The balloon attached to the inner tube is inflated to fix the transverse colon. (**H**) Outer tube reaches the entrance of the ascending colon. (**I**): Tip of the inner tube reaches the cecum in 340 s.

Figure 1B illustrates the insertion of the device based on our hypothesis. The inner tube was initially set inside the outer tube, and the lock/free mechanisms of the inner and outer tubes were set to free and lock, respectively. Figure 3 illustrates the first round of the insertion experiment. The device was inserted by hand from the anorectal side (Fig. 3A) and passed through the rectum by pushing and manipulating the tip of the outer tube (Fig. 3B). Insertion into the sigmoid colon is difficult by manipulating and bending the outer tube alone because the intestinal tract is highly bent. Therefore, the tip of the outer tube was set towards the insertion direction, the outer tube was fixed to the intestinal tract using a balloon, and the tip of the inner tube was pushed out. During pushing, the lock/free mechanism of the inner tube was set to lock and the bending of the inner tube was manipulated to avoid impinging on the folds of the canal wall (Fig. 3C). This procedure enabled the device to reach the descending colon without overstretching the wall of the colon model (Fig. 3D). To achieve this, the balloon attached to the inner tube was inflated and the outer tube was pushed forward to the position in which the inner tube was inserted, and the device was returned to the initial state (Fig. 3E). Subsequently, the entire device was pushed out and the tip reached the descending colon (Fig. 3F). Next, the tip of the inner tube was pushed out to reach the transverse colon (Fig. 3G). The outer tube was set again to its initial state (Fig. 3H). Finally, the tip reached the cecum owing to the pushing of the inner tube by bending and manipulating the outer tube (Fig. 3I). Pushing the inner tube required bending and manipulating the tip to pass through the folds of the colon model.

In three insertion experiments, a similar procedure was followed to achieve total insertion into the cecum of the colon model (see Supplementary Video S2 online). Insertion to cecum times is shown in Table 1. The average time required to pass through the sigmoid colon, which is highly bent, was approximately 43 s, and the average time to reach the cecum was approximately 442 s.

**Table1:** Result of colon model insertion time.

	Time to the sigmoid colon (s)	Time to the cecum (s)
1st	31	578
2nd	39	408
3rd	58	340

## Discussion

For insertion into the colon model, the maximum outer diameter of the device must be less than 20 mm. In this study, a DBE with a maximum outer diameter of 14 mm and outer and inner tubes that can bend independently and are electrically driven was molded using a 3D printer. Therefore, the mechanism of the developed device can be verified using the colon model. The experiments confirmed that the tip of each tube can bend without interference from wires and sheaths, the inflation and deflation of the balloons, and the pulling and pushing of the inner tube. Additionally, the inner tube can effectively bend and the balloon can be efficiently inflated/deflated, even when the outer tube experiences significant bending. Although, the payloads of the inner and outer tubes were 50 and 200 g, respectively, the weight of the device is considered to be sufficient to move through the several folds in the lumen because the weight of the colon model, which contains approximately 42 folds, was 379 g. Therefore, these structures achieved all the designed behaviors. No previous studies have verified the insertion of such a device structure into a colon model, and this study will contribute to the future development of robots for colonoscopy.

In advanced research, it has been reported that insertion into the colon model was investigated using developed robotic colonoscopes^14,22^. However, they have focused on validating the operation of the development mechanism; therefore, the colon model was replaced by simple materials such as a plastic wrap^14,22^ or simple intestinal tract arrangements obtained from an abdominal phantom^23^. However, these experiments differ from clinical colonoscopy, and whether these devices can pass through the flexible intestinal tract that is highly bent is unknown. In this study, a commercially available colon model used for colonoscopy training was used to simulate clinical intestinal tract arrangements using silicon rubber as a flexible material.

The insertion test indicated that the tip of the inner tube can follow the shape of the colon model by controlling the direction of the tip of the outer tube. The shape of the colon model was almost unchanged before and after insertion, thereby suggesting that our hypothesized motion allows for safe insertion without overstretching the colon. In addition, the developed system is intuitive as it can be operated electronically with a controller, and a non-medical operator can navigate the tip of the device to the cecum without the supervision of an endoscopist. Therefore, the learning curve of the device developed in this study for colonoscope insertion control is significantly lower than conventional colonoscopy systems.

Furthermore, insertion was tested for only one case (Case 1) in this study, and insertion into colon models with different shapes must be investigated in the future. The difficulty of insertion into colon models with other shapes increase because the device may encounter several highly bent structures in the sigmoid colon area. Particularly, the need of straightening the colon, which is an important step in conventional colonoscopy, has also been analyzed in the experiments. However, if the bending angle and minimum radius of the bending part are optimized, total colonoscopy can be expected owing to the motion described in Fig. 1B.

The purpose of this study was to determine whether the tip of the device could reach the cecum of the colon model with a clinically similar shape without over-stretching; therefore a translucent colon model was used to confirm the location of the tip from above. However, discerning the folds of the colon model externally without an endoscopic camera can be challenging. In some cases, particularly when colon bending is stronger than sigmoid colon in Case 1 at the hepatic and splenic flexures located at both ends of the transverse colon, the tip of the inner tube may impinge on the folds when the tube is pulled out because a camera has not been installed at the tip of the prototype. Approximately 442 s was required to reach the cecum. Yang et al*.*^23^ reported that the cecal insertion time was 354 ± 264 s for clinical colonoscopy. Hence, the target time should be within the cecal insertion time for any colon arrangement. Since the cecal insertion time of the colon model decreased with the number of attempts in this test, the time for insertion is expected to reduce with repeated operations. This suggests that the cecal insertion time can be shortened to avoid the folds of the colon model by visualizing the hollow cavity of the lumen using a camera. Although insertion could be performed without a camera using the transparent colon model in this experiment, sufficient colonoscopy experience is required to recognize insertion direction within the intestinal tract for clinical use. Therefore, image navigation functions, such as the detection of the direction of insertion combined with machine learning, as reported by Jiang et al*.*^24^*,* are necessary for colonoscopy to be performed even by a beginner. Although the camera was not implemented in this prototype, the inner tube was designed by assuming that a camera of dimensions equal to 1 mm^2^ (Osiris M Camera, Optasensor GmbH, Germany) and multiple optical fibers of 0.5 mm diameter as light guides will be included. These elements can be added for verification in the future. The operator using the proposed device is required to push and pull the flexible tube by holding it during insertion. However, to fully automate the insertion process, a mechanical function such as a continuum robot^25,26^ or robotic arm^27^ must be added.

In the current version of this device, safety features to prevent damage to the intestinal tract have not been implemented, as a colon model is being utilized. However, there is a potential risk of perforation due to balloon dilation or extrusion of the inner tube during the procedure. To mitigate this risk, it is essential to incorporate a feedback function that monitors the wire tension during bending, the pressure of the balloon, and the force exerted when pushing and pulling the flexible tube. These sensing requirements can be satisfied using a tension meter^28^ and tactile sensor arrays^29^. Additionally, accurately recognizing the shape of the inserted endoscope and the position of its tip is crucial for ensuring safety and navigating the insertion direction. To enhance safety, a system such as the Endoscope Position Detecting Unit (UPD) could be introduced, which provides a 3D image of the endoscope's shape and position within the body^30^. Incorporating these functions in future iterations of the device could enable even beginners and nurses to perform total colonoscopy procedures, such as screening and surveillance, through partially or fully automatic insertion.

However, there are certain limitations to consider. The device may be unsuitable for emergency endoscopy cases due to its differing shape and operation compared to conventional endoscopes. Furthermore, it may be more challenging to address occasional complications, such as bleeding or perforation, in comparison to conventional endoscopes. To manage these situations, it is necessary to include a forceps port that allows the use of conventional endoscopic instruments.

In conclusion, the developed device in this study can reach the cecum from the anus of the colon model without overstretching the model and can be operated by a single non-medical operator. This suggests that the proposed prototype can facilitate a comfortable colonoscopy.

## Materials and methods

### Design of the outer and inner tubes

**Figure 4:**
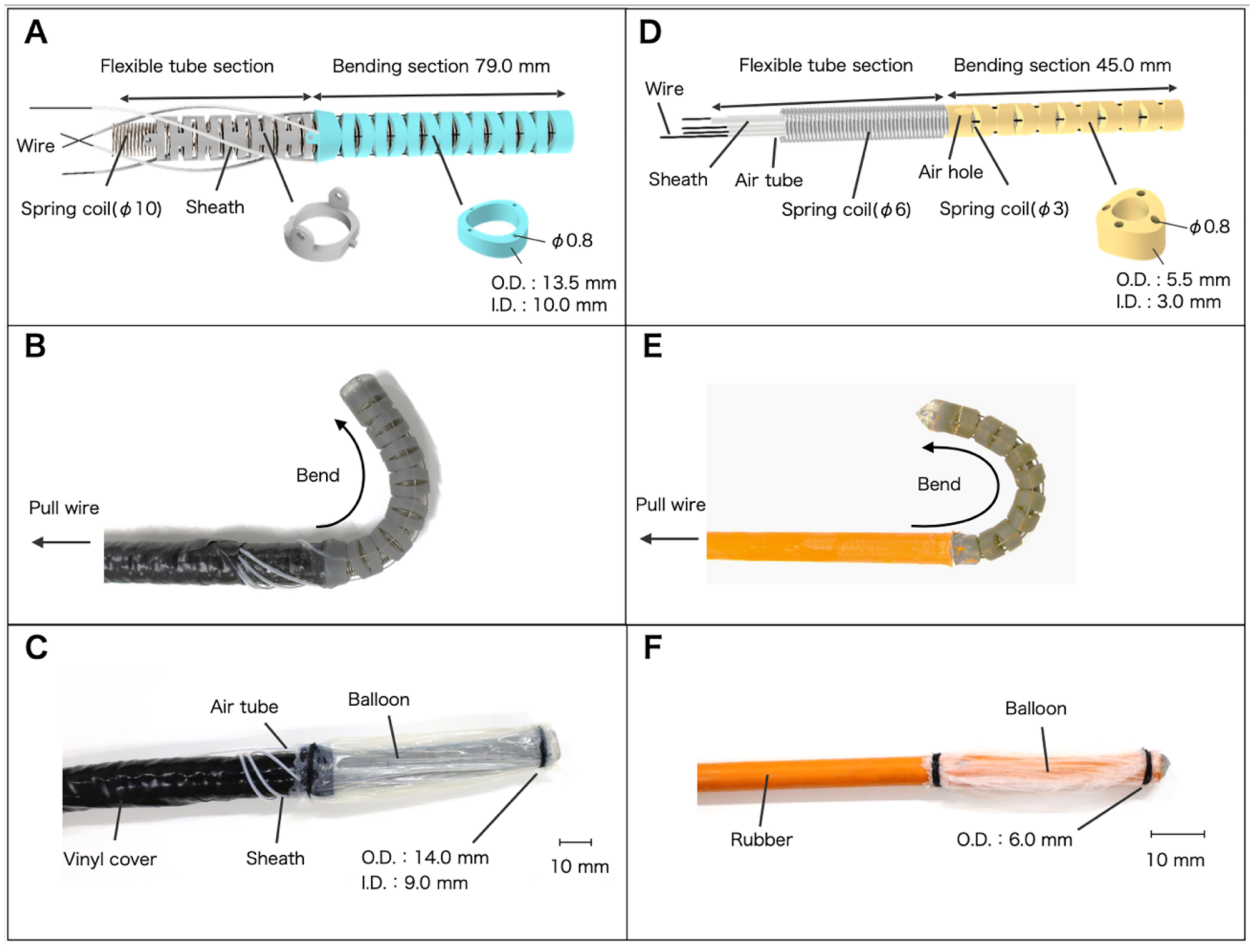
(**A**) Segment of the outer tube designed for insertion. (**B**) 3D-printed components and bending motion in the bending section of the outer tube. (**C**) Fully assembled outer tube. (**D**) Segment of the inner tube designed for insertion. (**E**) 3D-printed components and bending motion in the bending section of the inner tube. (**F**) Fully assembled inner tube.

The outer and inner tubes with attached balloons, which can be manipulated electrically, were developed. The frame of the outer tube was composed of two types of bending parts manufactured using a photocurable resin (Graypro, Formlabs Co., USA), 3D printer (Form 3, Formlabs Co., USA), and coil (L041, Accurate Inc., Japan) with an outer diameter (OD) of 10 mm, to be passed through the parts as shown in Fig. 4A. The parts of the bending section were assembled to possess a diameter of 13.5 mm and a total length of approximately 79 mm, and the 3D-printed parts (light blue colored parts in Fig. 4A) were assembled was bent using wire traction^31^. The parts of the flexible tube section were assembled with 3D-printed parts (gray colored parts in Fig. 4A), which are rotated by 90 degrees and fit together. The wires (SB-036-50M, Osaka Coat Rope Co., Ltd., Japan) were passed through a sheath (TUF-100-AWG-26-10M, Chukoh Chemical Industries, Ltd., Japan) outside the flexible tube section and arranged in a spiral shape to avoid interference with each other during bending. The frame of the flexible tube section and sheath was coated with vinyl chloride. The bending motion is illustrated in Fig. 4B. The bending section was covered with a double layer of latex rubber (Latex tube, Fuji Latex Co., Ltd., Japan), and an air tube (TUF-100-AWG-17-10M, Chukoh Chemical Industries, Ltd., Japan) was placed between the layers to inflate and deflate the layer as a balloon, as shown in Fig. 4C. As shown in Fig. 4D, the inner tube consists of a flexible tube and bending sections. The bending section is assembled using 3D-printed parts with an OD of 6 mm (orange colored parts in Fig. 4D) that have through holes for wires and has a coil (L006, Accurate Inc., Japan) with an OD of 3 mm inside to increase stiffness. The flexible tube section has a coil with an OD of 6 mm, an air tube (TUF-100-AWG-17-10M, Chukoh Chemical Industries, Ltd., Japan), and four sheaths (TUF-100-AWG-26-10M, Chukoh Chemical Industries, Ltd., Japan) to thread the wires through. The bending motion is Tillustrated in Fig. 4E. The entire length of the tube is covered with rubber (Pencil balloon, Suzuki Latex Co., Ltd., Japan) and the bending section has a balloon (Latex balloon, Fuji Latex Co., Ltd., Japan), as shown in Fig. 4F. The junction of the flexible tube and the bending part has an air hole, and an air tube is connected to inflate and deflate the attached balloon.

### Drivers of the outer and inner tubes

**Figure 5:**
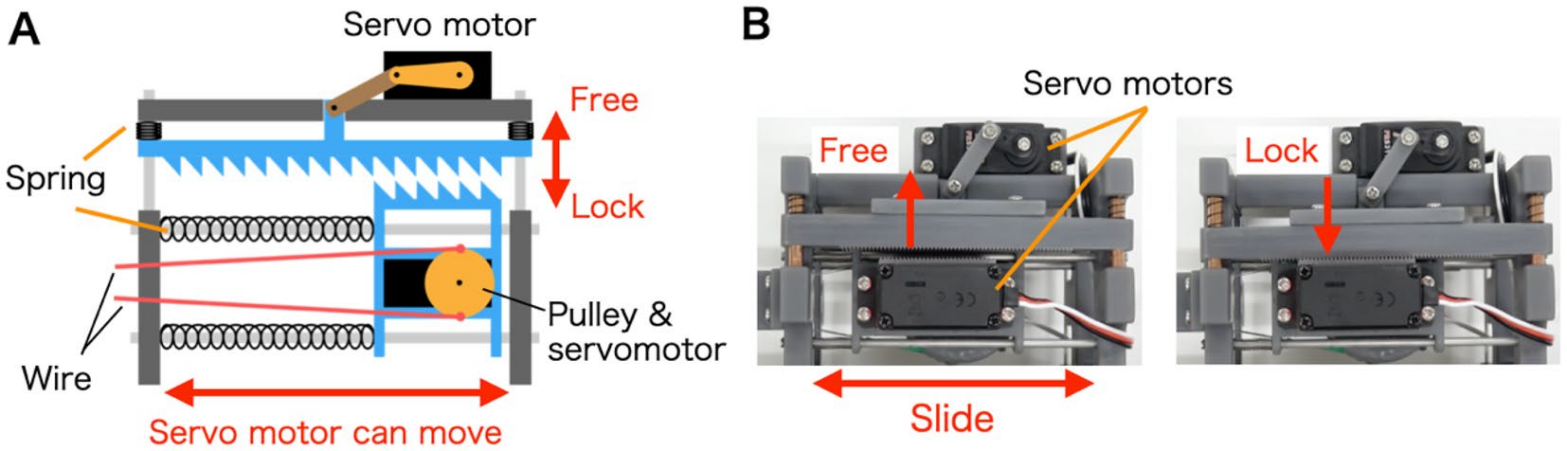
(**A**) Schematic design of the lock/free mechanism for positioning the servomotor. (**B**) Motion of the servomotor.

**Figure 6:**
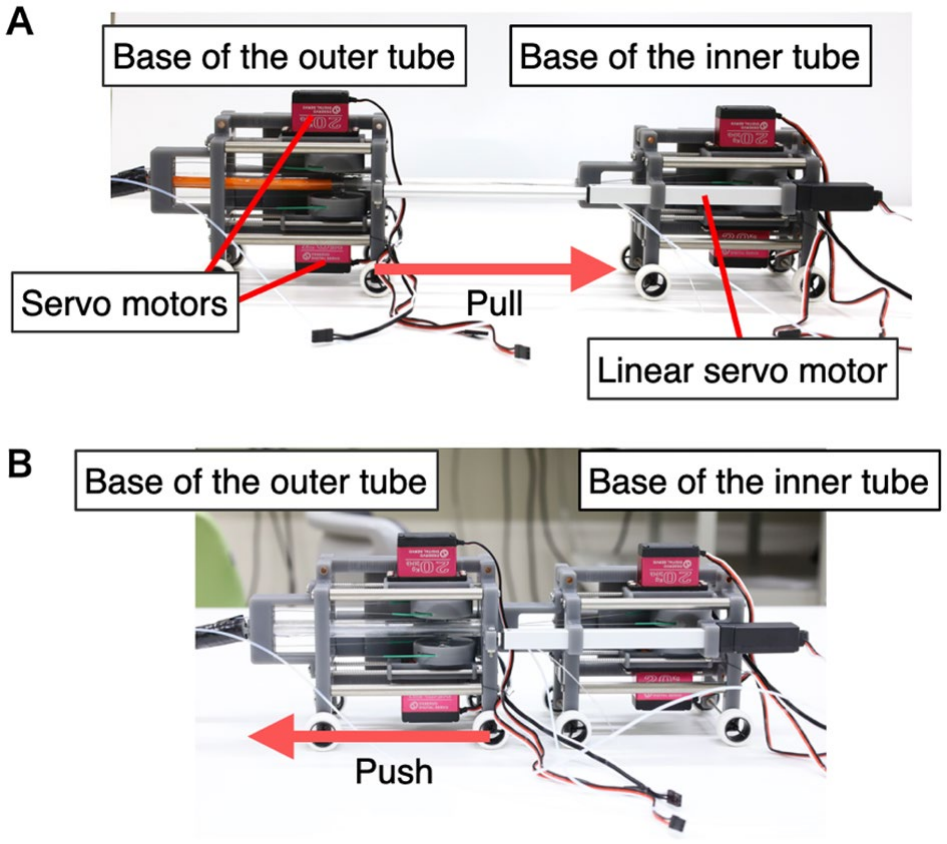
Pulling and pushing mechanism of the inner tube using a linear servomotor. (**A**) Pushing motion. (**B**) Pulling motion.

The drivers of the outer and inner tubes consist of three servomotors that have a pulley for up/down and left/right traction (DS3218 (270° ver.), Goolsky, China), as well as the lock/free mechanism (FR5311M, FEETECH RC Model Co. Ltd., China). As shown in Fig. 5A, the lock/free mechanism can position the servomotors that have a pulley slide to balance the pull exerted on the wire and reactive force of the springs on the rail. The motion can release fixation using wire tension when the flexible section is bent. The frames of the basic structures are constructed using a 3D printer, as shown in Fig. 5B. Additionally, the bases of the outer and inner tubes are connected via a linear servomotor with a 140-mm stroke (Actuonix L16, Actuonix Motion Devices Inc., Canada). As shown in Fig. 6A, the shaft is initially fully extended, and the tip of the inner tube is pulled into the outer tube. By shortening the shaft, the inner tube is pushed out from the tip of the outer tube, as shown in Fig. 6B. In this study, these operations were verified, and the maximum loading capacities of the bending sections were investigated.

### Controller system

**Figure 7:**
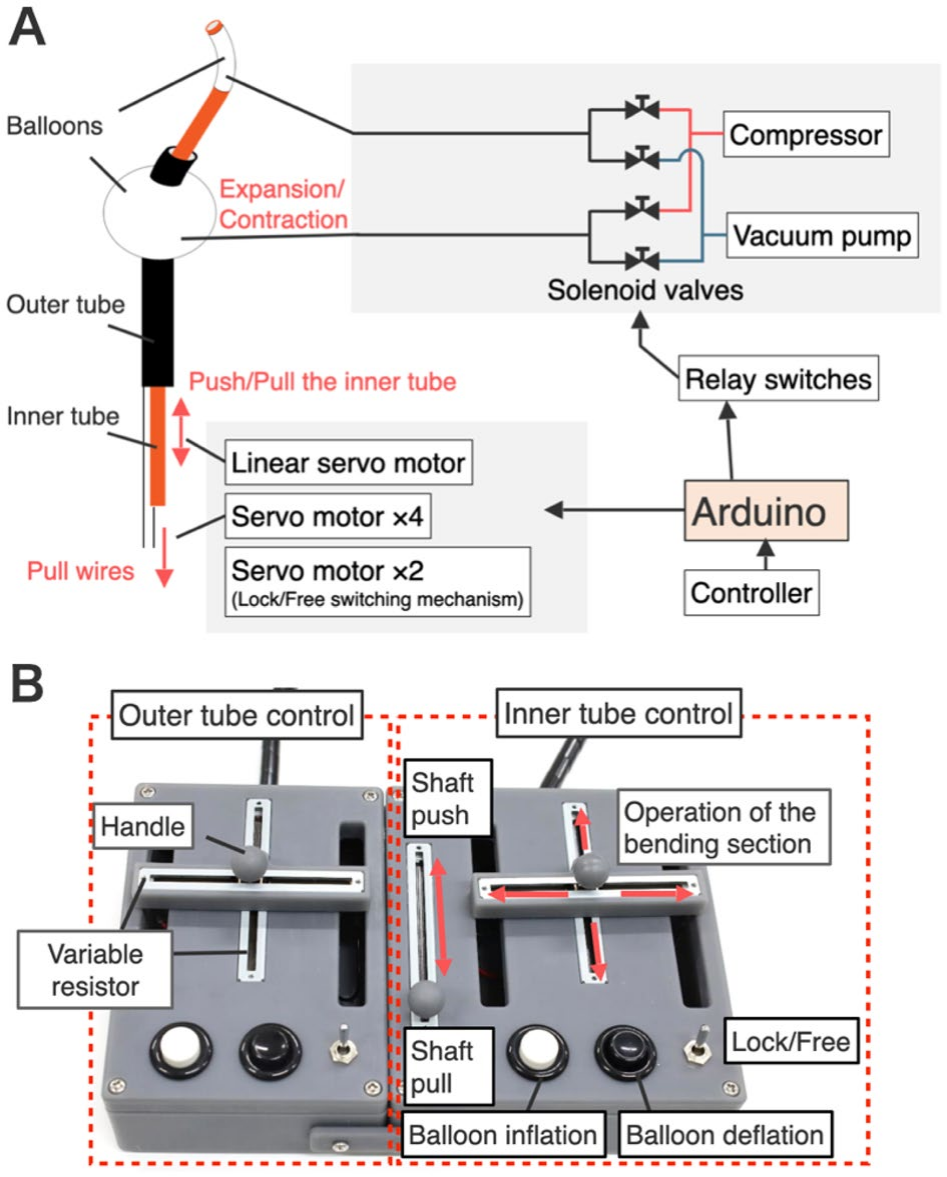
Development of the device control system (**A**) Overall architecture of the device. (**B**) Controller of the device.

To control all the electrical drives, the motors and relay switches were connected to an Arduino Mega 2560 R3, as shown in Fig. 7A, and the controller was designed, as shown in Fig. 7B. The shaft length of the linear servomotor (0–140 mm) and the displacement angles (0–180º) of all the servomotors for driving the bending motion were determined using the resistance value of the sliding-type variable resistor (RS6011Y19004, ALPS ALPINE CO., LTD., Japan). The variable resistor controlling horizontal motion was connected to the knob of another variable resistor controlling vertical motion. Accordingly, vertical and horizontal bending was achieved via a single knob. The lock and free states were assigned by reading the toggle switch ON/OFF as a digital signal, and the displacement angle (0 or 40º) of the servomotor is set. The inflation and deflation of each balloon were controlled using white and black button switches, respectively, and these, in turn, were controlled by relay switches that open or close solenoid valves connected to a compressor(ACP-10A, Takagi Co., Ltd., Japan) and vacuum pump(DA-40S, ULVAC KIKO, Inc., Japan).

### Verification of the insertion of the device using a colon model

To verify the insertion of the developed device into a colon model, a translucent colon model was used (Colonoscope training model, KYOTO KAGAKU Co., LTD, Japan). The colon was set in the shape of Case 1, and lubricant was used during insertion. One engineer (non-medical) operated the device by checking the position of the device tip directly from the top of the transparent colonoscope model. This insertion operation was repeated three times.

## Supplementary Information

Supplementary Legends.

Supplementary Video 1.

Supplementary Video 2.

## Data Availability

The datasets generated in this study are available from the corresponding author upon reasonable request.
