# Supplementary material for: Robotic endoscope with double-balloon and double-bend tube for colonoscopy
Source: Sci Rep. 2023 Jun 28;13:10494. doi: 10.1038/s41598-023-37566-3 (PMC10307855; doi:10.1038/s41598-023-37566-3)
Supplement: Supplementary file 1 — Supplementary Legends. [file 41598_2023_37566_MOESM1_ESM.docx]

**Supplementary Information**

**Supplementary Video S1.** Demonstration video of the developed device

**Supplementary Video S2.** The fastest insertion experiment video of this study
